# The mediating effect of resilience between family functioning and mental well-being in hemodialysis patients in Japan: a cross-sectional design

**DOI:** 10.1186/s12955-020-01486-x

**Published:** 2020-07-17

**Authors:** Hiroko Kukihara, Niwako Yamawaki, Michiyo Ando, Midori Nishio, Hiromi Kimura, Yoshiko Tamura

**Affiliations:** 1 Fukuoka University Faculty of Medicine, School of Nursing, 7-45-1 Nanakuma, Jonan-ku, Fukuoka, 814-0180 Japan; 2 Department of Psychology, Brigham Young University, 1094 KMBL, Provo, UT 84162 USA; 3 St. Mary’s College, School of Nursing, 422 Tsubuku-honmachi, Kurume, Fukuoka, 830-8558 Japan; 4 Department of Nursing, Yasuda Women’s University, 6-12-1 Yasuhigashi, Asaminami-ku, Hiroshima, 731-1153 Japan

**Keywords:** Resilience, Family cohesion, Adaptability, Communication, Mental well-being, Dialysis patients

## Abstract

**Background:**

End-stage kidney disease is highly prevalent worldwide. Currently, one of the most effective treatment modalities is dialysis therapy, which leads to serious side effects. Furthermore, psychiatric illnesses are prevalent among dialysis patients. Recently, researchers asserted that psychological resilience and family support could be helpful to maintain or improve patients’ mental well-being. Therefore, the purpose of this study was to examine the mediating effects of resilience on the relationship between family functioning and mental well-being in these patients.

**Methods:**

To investigate the aim of this study, a cross-sectional design was employed. A total of 110 hemodialysis patients, who were receiving outpatient treatment from dialysis units at the University of Fukuoka and St. Maria Health Care Center in Japan, participated. Only the patients who met the criteria and who were willing to participate in this 30-min study were given The General Health Questionnaire-12, Conner-Davidson Resilience Scale, and Family Assessment Device. Structural Equation Modeling (SEM) was performed to test the hypothesis that resilience would mediate the relationship between each subscale of family functioning, namely, cohesion, adaptability, communication, and mental well-being. Then Sobel’s test was employed to examine the indirect effect.

**Results:**

The results of the SEM showed that the model had an acceptable fit (RMSEA = .077; CFI = .93; and IFI = .94). According to the results, resilience fully mediated the relationship between family functioning, specifically family adaptability and communication, and mental health well-being of the dialysis patients. However, family cohesion was not associated with resilience.

**Conclusions:**

The present study revealed that higher family adaptability and communication resulted in greater resilience, thus associated with better mental health. Given that poor mental health among dialysis patients is significantly associated with a decreased likelihood to adhere to treatment plans, it may lead to a significant risk to therapeutic compliance. As such, patients may experience detrimental consequences, such as death. This study showed that in order to maintain healthy mental well-being, developing resilience is a vital factor for hemodialysis patients.

## Introduction

Kidney disease has become one of the major threats to humanity’s health worldwide. It not only has indirect impact on global morbidity and mortality [1], but it also is associated with enormous economic burden [2]. According to Wang et al. [3], 1.2 million people died from kidney failure in 2015, a 32% increase since 2005. An estimated 2.3 to 7.1 million people with end-stage kidney disease (ESKD) died in 2010 without access to dialysis. Furthermore, because this disease is associated with increased risk of deadly diseases, such as cardiovascular disease, diabetes, and hypertension [4], it is crucial to have effective prevention programs and access to treatments.

In Japan, ESKD is highly prevalent, especially among elderly patients in larger populations [5]. According to the Japanese Society for Dialysis Therapy, one in 378.8 Japanese have ESKD [6, 7] Fortunately, though the prevalence of ESKD is extremely high in Japan, almost all Japanese individuals who need dialysis can receive the treatment [8]. As such, Japan has become one of the most prevalent users of dialysis therapy in the world, and more than 230,000 patients are on dialysis therapy today [8]. Dialysis therapy is effective, but there are serious side effects reported. Murtagh et al. [9] found that dialysis patients suffer from lack of energy, pruritus, drowsiness, dyspnea, edema, pain, dry mouth, muscle cramping, restless legs, lack of appetite, poor concentration, dry skin, sleep disturbance, and constipation. Patients on dialysis must abjectly depend on a machine for the rest of their lives and struggle with these adversarial physical symptoms. Furthermore, they must continuously adhere to dietary and fluid restrictions for the rest of their lives [10]. Such restrictions may lead patients to be socially isolated because of their need for time-consuming dialysis treatment to successfully manage ESKD [10]. Additionally, they suffer from loss of employment, significant economic burden [11], negative body image, and sexual dysfunctions [12].

Predictably, psychiatric illnesses, such as depression and anxiety, are rampant among these patients. According to Couch, almost every patient with ESKD experiences depression at one stage or another [13] Yet, some patients are able to adjust and maintain good mental health. The effects of resilience have been garnering a great deal of attention and may help to explain the differing mental-health outcomes of those who experience adversarial and/or traumatic events. Resilience is a protective factor that buffers from the effects of traumatic experience. It is a personality characteristic that enhances individual adaptation and positively influences successful adaptation and coping [14]. The effects have been well documented for disaster survivors [15], patients with HIV [16], war veterans with PTSD [17], and so on. Researchers assert that personal factors such as resilience [15] and family support [18] could explain the differences in mental-health outcomes. Good relationships among family members is significantly associated with emotional comforts and greatly influence life quality [19]. Therefore, in the present study, the effects of such personal factors were investigated to explain the possible differences in patients’ mental-health outcomes. In particular, the purpose of this study was to examine the mediating effects of resilience on the relationship between family functioning and perceived physical and mental health. Evidences have shown, in studies using meta-analysis and a large sample, that physical exercise improve mental health [20, 21]. However, Patients with ESKD experience substantial loss of muscle mass, weakness, and poor physical performance. They further experience mobility limitation, loss of functional independence as kidney disease progresses, which may lead them to limit exercising [22]. This study is noteworthy to investigate since examining the mediating role of resilience between family support and perceived physical/mental health might lead to improve their perceived physical/mental health without engaging in physical exercise. To the best of our knowledge, this is the first study to explore such relationship using patients with ESKD.

## Methods

### Study design and participants

This study took place at hemodialysis units at the University of Fukuoka and the St. Maria Health Care Center in Japan. These hospitals were selected because their administrators granted permission and provided assistance to collect data. University of Fukuoka holds 25 beds and 50 patients come to receive treatment weekly. Among them, 38 (76%) patients agreed to participate in this study. However, the data from nine participants were dropped due to being unable to complete the survey. St. Maria Health Care Center has 63 beds and 126 patients visit the center weekly. Among them, 95 (75.4%) patients agreed to participate in this study, but the data from 12 participants were dropped. Therefore, a total of 110 hemodialysis patients (male = 72; female = 38; mean age = 64.5) participated. Their sociodemographic information is summarized in Table 1. Doctors and nurses of the dialysis units selected patients based on the inclusion and exclusion criteria. Participants in the present study were patients undergoing hemodialysis treatment who were 20 years of age or older and who were outpatients living with their family members. Patients who had dementia, who were unconscious, or who were unable to successfully complete the survey were excluded from the study. Research collaborators (nurses in the units) explained details of the study to potential eligible participants. Only the patients who met the criteria and who were willing to participate in a 30-min study were given the survey. Upon completion, they were asked to place the survey in an envelope and put it in a designated box outside of the hemodialysis units. Study participants were not compensated, and their participation was completely voluntary. The study protocol was approved by the Ethics Committee of Fukuoka University, and informed, written consents were obtained from all participants.

**Table 1:** Demographic characteristics

		*n* = 110
Demographic	n	(%)
Gender		
Male	72	(65.5)
Female	38	(34.5)
Age (years) Mean ± SD,Range	64.3 ± 11.5, 31 - 86	
Family living arrangement		
Married couple with no children	32	(29.1)
Married couple with children	31	(28.2)
Three generations	13	(11.8)
Married couple with parents	8	(7.3)
Married couple with unmarried children	11	(10.0)
Other	11	(10.0)
Unanswered	4	(3.6)
History of dialysis (month) Mean ± SD,Range	160.4 ± 107.0, 14 - 512	
Occupation		
Paid worker	26	(23.6)
Self-employed	18	(16.4)
Non-employed	52	(47.3)
Homemaker	10	(9.1)
Unanswered	4	(3.6)
Economic condition		
Very meager	3	(2.7)
Meager	7	(6.4)
Normal	58	(52.7)
Comfortable	6	(5.5)
Very comfortable	1	(.9)
Unanswered	35	(31.8)
Disease		
CGN; chronic glomerulonephritis	35	(31.8)
DM nephropathy	36	(32.7)
Other	39	(35.5)

### Measurements

#### The general health Questionnaire-12 (GHQ-12)

The GHQ-12 has been widely used in a variety of settings across countries, and its validity and reliability have been established in Japan with internal consistency of .84 and yielding two-factor structures, which is similar to the psychometric properties of the original study [23]. It is a self-administered screening questionnaire for the purpose of identifying minor psychiatric disorders in the general population, and it is used within the community and with non-psychiatric individuals. This scale is designed to assess respondents’ general mental health over the previous 4 weeks, and respondents were asked to rate their levels of happiness, depression, anxiety, and sleep disturbance. Each item is scored as 0 (*less or no more than usual*) or 1 (*rather or much more than usual*), giving a possible range of the score 0 to 12. Scores above a threshold of 4 or more are regarded as indicating psychiatric morbidity. This approach has been recommended by the developer of this scale [24] and has been shown to be applicable to the Japanese version [25]. The internal consistency for the current study was .86.

#### Conner-Davidson resilience scale (CD-RISC)

The CD-RISC was developed to assess an individual’s ability to cope with traumatic stress [26]. This measure has been widely used in cross-cultural research to objectively quantify resilience, and the reliability and validity of this scale have been verified in numerous studies [27, 28]. It contains 25 items, all of which have a 5-point Likert scale response, ranging from 0 = *not true at all* to 4 = *true nearly all of the time*. The scale is rated based on how the respondent has felt over the past month. The total score ranges from 0 to 100, with higher scores reflecting greater resilience. The Cronbach’s alpha of this scale in the present study was .94.

#### Family assessment device (FAD)

The FAD is a 60-item questionnaire and was developed based on the McMaster model of family functioning [29]. It is designed to collect information on various structural and organizational dimensions of the family system. This questionnaire is a self-administered measure that assesses family functioning and quality of interaction among family members. Respondents rate on a 5-point Likert scale: *very rarely, rarely, occasionally, frequently,* and *very frequently*. The validity and reliability of the FAD have been established in the United States. However, although the FAD has been used in many countries, factor analysis shows that it is a poor fit for non-Caucasian ethnic minority respondents [30]. The Japanese-translated version of the FAD also shows many challenges that prevent it from establishing its validity and reliability [31]. As such, Takemoto and Kagawa [32] suggest using three subscales, namely, cohesion, adaptability, and communication, when the study’s purpose is to investigate the relationship between family functioning and patients’ mental well-being. They concluded that these three subscales were quick and effective tools to assess family functioning. Adaptability is defined as the ability of the family system to change when changing the situation is required, and the subscale measures the degree to which the family can tolerate such changes. A previous study showed that family members who are adaptable tend to tolerate changes, as required by the family life cycle, and adaptable families seem to be the best family system for people who have experienced traumatic events. Therefore, adaptability may impact on one’s mental health well-being. A typical item of the Adaptability subscale is: “We know what to do when an emergency comes up.” (“Watashino uchideha kinkyuujitaini dousurebayoika siteteiru.”). Cohesion is characterized by emotional bonding between family members. Highly cohesive families tend to unite to resolve their problems and provide mutual support, and cohesion may impact positive mental health well-being. An example of an item (reversed item) for the Cohesion subscale is: “We are too self-centered in our family.” (“Watashino uchideha minaga amarinimo jikochushintekida.”). Finally, Communication refers to the way verbal and non-verbal information is exchanged between family members, and this subscale assesses the degree to which family members’ are capable of listening and sharing feelings and being clear, focused, and respectful regarding the other family members. Black and Lobo [33] suggested that clear and open communication among family members during a traumatic event can improve their mental health. Thus, higher communication may impact one’s mental health among hemodialysis patients. One of this subscale items (reversed item) is: “We avoid discussing our fears and concerns.” (“Watashino uchideha osoreteirukotoya shinpaigotoni tsuite hanashiaunowo saketeiru.”). Therefore, for the purpose of this study, and to reduce the burden on hemodialysis patients who can become tired when answering the original 60-item FAD, we employed Takemoto and Kagawa’s suggestion and gave 12 items having to do with these subscales. The internal consistencies for the cohesion, adaptability, and communication subscales were .85, .81, and .82, respectively.

### Data analyses

**Fig. 1:**
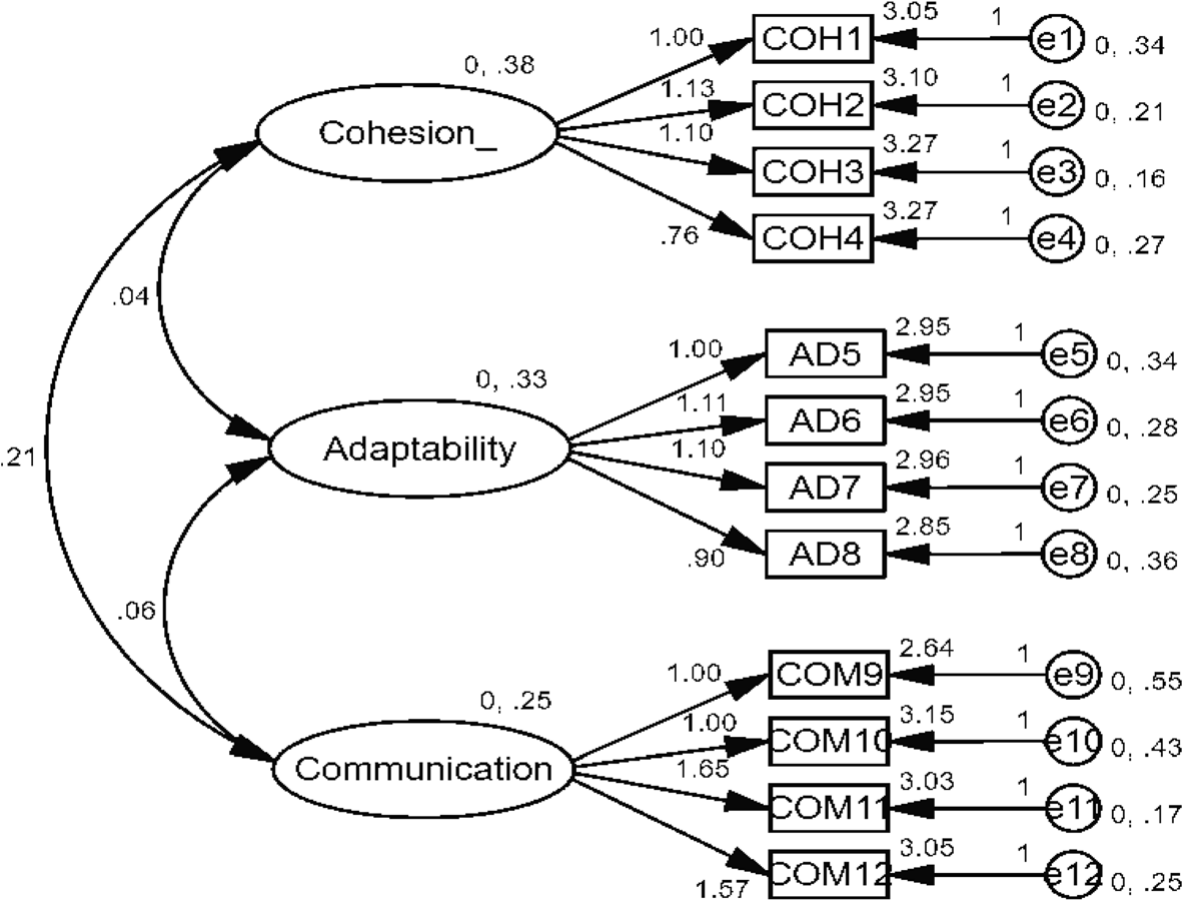
The measurement model. All factor loadings are unstandardized with *p* < 0.001. Observed variables of Cohesion: COH1 = Self-centered; COH2 = Unsupportive; COH3 =Uninterested; COH4 = Disregard family rules. Observed variables of Adaptation: AD5 = Adaptive under emergency; AD6 = Take family responsibilities; AD7 = Solve problems; AD8 = Face issues in family. Observed variables of Communication: COM9 = Express love; COM10 = Avoid fear; COM11 = Express tender feeling; COM12 = Show no love.

Although GHQ and CD-RISC have been extensively used in Japan, the FAD has not been widely used. Thus, to evaluate the fit of the FAD to Japanese hemodialysis patients, a confirmatory factorial analysis (CFA) was performed and the model fit was evaluated by means of structural equation modelling (SEM) using AMOS v26. Having a large-sample technique is recommended to use SEM [34]. However, since it is extremely difficult to obtain a large sample of hemodialysis patients who met all inclusion criteria, a model is said to fit when the Comparative Fit Index (CFI) and Incremental Fit Index (IFI) are higher than .9. Additionally, the root mean-square error of approximation (RMSEA) must be below .08 [35]. The descriptive analyses were performed using the software package SPSS v26. In order to determine whether the theoretical models fit or not, SEM was employed using AMOS 26 (see Fig. 1). All variables, such as adaptability, cohesion, communication, resilience, and mental health well-being were included. Finally, to test the indirect effect, Sobel’s test [36] was performed.

## Results

Descriptive statistics, reliability estimates (Cronbach’s alpha coefficients), and correlations for all the study variables are presented in Table 2.

**Table 2:** Means, standard deviations (SD),reliabilities aand intercorrelations among study variables

Measure	Mean	SD	α	1	2	3
1.Cohesion	12.69	2.66	.85	1		
2.Adaptability	11.75	2.64	.82	.097	1.000	
3.Communication	11.77	2.94	.81	.578**	.291**	1
4.Resilience	82.42	17.26	.94	.251**	.235*	.334**
5.GHQ	3.69	2.71	.76	-.317**	-.271**	-.354**

* = *p* < .05; ** = *p* < .01

### Measurement model

The measurement model comprised of three latent factors (Cohesion, Adaptability, and Communication) and 14 observed variables showed acceptable fit to the present data:

RMSEA = .086; CFI = .93; and IFI = .93. All the factor loadings for the indicators on the latent variables were reliable (*p* < .0001), indicating that all the latent factors were well represented by their respective indicators.

### Structural model

**Fig. 2:**
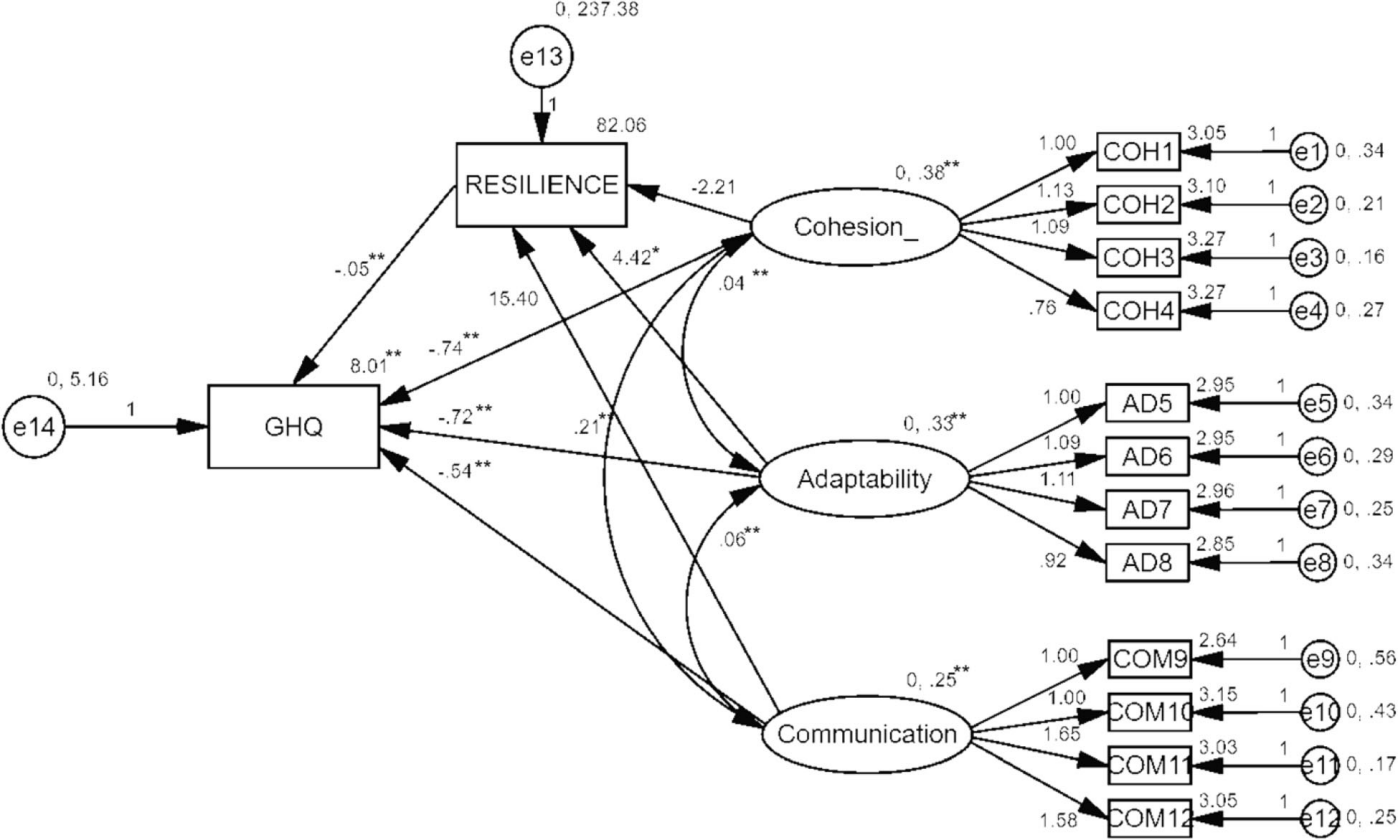
Final causal model. Values represent unstandardized factor loadings and standardized regression estimates. * = *p* < .05; ** = *p* < .01. Observed variables of Cohesion: COH1 = Self-centered; COH2 = Unsupportive; COH3 = Uninterested; COH4 = Disregard family rules. Observed variables of Adaptation: AD5 = Adaptive under emergency; AD6 = Take family responsibilities; AD7 = Solve problems; AD8 = Face issues in family. Observed variables of Communication: COM9 = Express love; COM10 = Avoid fear; COM11 = Express tender feeling; COM12 = Show no love. GHQ = General Health Questionnaire.

To investigate the influence of family functioning, namely, cohesion, adaptability, and communication, on mental health well-being and the possible mediation role of resilience, the structural model was examined using maximum likelihood estimation. This analysis was employed again with AMOS v26 to estimate the direct paths depicted in Fig. 2. The mediation model of resilience in the relationship between family functioning and mental health demonstrated an acceptable fit to the data, with RMSEA = .077; CFI = .93; and IFI = .94. The SEM results showed significant direct effects of family functioning (cohesion, adaptability, communication) on mental health (b = −.74, *p* < .01; b = −.72, *p* < .01; b = −.54, *p* < .01, respectively) and resilience (b = − 2.21, *ns*; b = 4.42, *p* < .05; b = 15.4, *p* < .01, respectively). The SEM results also showed a significant effect of resilience on mental health (b = −.05, *p* < .0001). Sobel’s test was then conducted to test the significance of a mediation effect. Since the relationship between cohesion and resilience was not significant, Sobel’s test was not employed for its indirect effect. Resilience significantly mediated the links between family functioning (adaptation and communication) and mental health well-being (z = 2.14, *p* < .05; z = 2.74, *p* < .01, respectively).

## Discussion

The aim of this study was to investigate the effect of resilience on the relationship between family functioning and mental well-being of hemodialysis patients in Japan. Greater resilience and higher family functioning have been shown to be associated with better mental well-being. This study revealed that family functioning affects the resilience and mental well-being of hemodialysis patients.

As for the link between family functioning and mental well-being, in line with our hypothesis, participants who perceived that their family members can express their affection to each other, are able to discuss fears and concerns with each other, and can talk about their tender feelings tended to show better mental well-being. Furthermore, participants who perceived that their family members are highly adaptable, such as having clear duties and responsibilities in their family so that they can make decisions to solve problems effectively and know what to do in cases of emergencies, showed better mental well-being. In addition, greater family cohesion was associated with better mental well-being. That is, participants who see their family as being less self-centered showed more interest in them and were more involved in each other’s lives tended to show better mental health. One of the most important tasks for hemodialysis patients is to adhere to a highly invasive non-curing treatment, which involves a specific diet, restricted fluid consumption, and high cost of medication intake [37]. Because such restriction is extremely difficult for the patients, low adherence to this treatment is well documented [38]. In particular, hemodialysis patients suffering from mental illness and stress are negatively associated with adherence to treatment; thus, there is a significant risk to their therapeutic compliance [39]. For instance, a depressed patient becomes non-adherent to the treatment 176% times more than a non-depressed patient [40]. This lack of compliance to the treatment has detrimental consequences, especially for patients over 65 years old, given that their life expectancy is less than 5 years after dialysis initiation [41]. Therefore, maintaining healthy mental well-being is of vital importance for dialysis patients. To do so, it is strongly recommended to assist family members of dialysis patients to foster cohesive, adaptive and communicative functioning.

The results of this study further revealed the mediating role of resilience on the link between family functioning and mental health of the dialysis patients in Japan. Specifically, having adaptive and communicative family functioning was associated with greater resilience which was resulted in having better mental health. When hemodialysis patients perceive that their family members are able to adapt well in adversarial and traumatic events, are able to express love and trust, and have good problem-solving skills, they tend to show stronger resilience. Such relationships create love and trust, and they may provide encouragement and reassurance that help bolster patients’ resilience [42]. This study added to the current literature that there is crucial influences of family adaptation and communication on resilience and mental well-being. Dialysis is a complex treatment procedure that leads to significant changes in the patients’ lives and to their increased dependence on the caregivers, who are frequently family members. As a result, caregivers of dialysis patients are inclined to experience a significant burden, which has an adverse effect on their own quality of life due to patients’ overreliance on family members who themselves may not have necessary support systems [43]. Such lack of support results in stress on the family system and on the caregivers’ physical, mental, and social health. Families are one of the indispensable caregiving resources for dialysis patients and have a fundamental role in managing the patients’ diseases and in improving their mental well-being [44]. Therefore, care and support are required, not only for dialysis patients, but also for family caregivers. Multidisciplinary team intervention by professionals such as physicians, nurses, mental-health service providers, and so on, may be necessary to offer effective care and treatments for dialysis patients and their family members. Such interventions may play a critical role in improving the physical and mental health of dialysis patients and their family members. In addition, because family adaptability and communication influences a patient’s resilience, family or couple therapy may be extremely beneficial by addressing communication skills and by building trust and problem-solving skills.

In addition, the results of this study also indicated that building resilience is also an effective way to improve the mental health of dialysis patients in Japan. In fact, Bahremand et al. [18] suggested that resilience is more important than family functioning when mental health is concerned, and mental health is affected by resilience rather than family functioning [19]. Therefore, we suggest that the primary focus of caregivers should be encouraging resilience in dialysis patients. Previous studies have found that the primary contributor to resilience is the relational basis of resilience—resilience results from having caring and supportive relationships [45]. However, there is some evidence that resilience is strongly associated with non-relational bases. For instance, Kukihara et al. found that physical exercise promotes resilience among elderly individuals [46]. Furthermore, being employed and having a healthy lifestyle, including a good diet and enough sleep, were significantly associated with greater resilience [15]. Resilience can be achieved not only through relational bases, such as family relationships, but also through non-relational bases, such as good sleep, exercise, and healthful eating habits. Building resilience is a dynamic process, and interventions such as mindfulness-based skills and cognitive-behavioral approaches could be utilized to dialysis patients in order to increase the protective effect of resilience on their mental health [47, 48]. It is also recommended to regularly engage in a proactive personal reflective report to increase their resilience [48]. Overall, intervention strategies that are specifically feasible to dialysis patients should be conducted in future research. For another future study, it is vital to investigate the relationship between perceived mental health well-being and the existence of comorbid conditions in hemodialysis patients, such as heart failure, coronary heart disease, lung disease, cerebrovascular disease, and psychiatric disorders, since comorbidity of other diseases are very common among hemodialysis patients. Comorbid conditions may be likely to affect the self-reported mental well-being; nevertheless, this study did not explore such relationship.

## Conclusion

The present study found mediating effects of resilience on the relationship between family functioning (adaptability and communication) and mental well-being among hemodialysis patients in Japan. That is, higher family adaptability and communication were associated with greater resilience, which was related to better mental health. Given that poor mental health among dialysis patients is significantly associated with less likelihood to adhere to demanding treatment plans, dialysis patients with poor mental health pose a significant risk to therapeutic compliance. As a result, these patients may experience detrimental consequences; patients over 65 years old are especially at risk, given that their life expectancy is less than 5 years after dialysis initiation. This study showed that developing resilience is one of the vital factors for hemodialysis patients to maintain healthy mental well-being.

## Data Availability

Please address requests to hkukihara@adm.fukuoka-u.ac.jp.
